# Influence of Small Amounts of ABS and ABS-MA on PA6 Properties: Evaluation of Torque Rheometry, Mechanical, Thermomechanical, Thermal, Morphological, and Water Absorption Kinetics Characteristics

**DOI:** 10.3390/ma15072502

**Published:** 2022-03-29

**Authors:** Carlos Bruno Barreto Luna, Edson Antonio dos Santos Filho, Danilo Diniz Siqueira, Edcleide Maria Araújo, Emanuel Pereira do Nascimento, Tomás Jeferson Alves de Mélo

**Affiliations:** Academic Unit of Materials Engineering, Federal University of Campina Grande, Av. Aprígio Veloso, 882-Bodocongó, Campina Grande 58429-900, Brazil; edson.a.santos.f@gmail.com (E.A.d.S.F.); danilodinizsiqueira@gmail.com (D.D.S.); edcleide.araujo@ufcg.edu.br (E.M.A.); emanueluepb@gmail.com (E.P.d.N.); tomas.jeferso@ufcg.edu.br (T.J.A.d.M.)

**Keywords:** polyamide 6, ABS, ABS-MA, polymer blends, tailoring of properties

## Abstract

In this work, polyamide 6 (PA6) properties were tailored and improved using a maleic anhydride-grafted acrylonitrile-butadiene-styrene terpolymer (ABS-MA). The PA6/ABS-MA blends were prepared using a co-rotational twin-screw extruder. Subsequently, the extruded pellets were injection-molded. Blends were characterized by torque rheometry, the Molau test, Fourier transform infrared spectroscopy (FTIR), impact strength, tensile strength, Heat Deflection Temperature (HDT), Differential Scanning Calorimetry (DSC), Thermogravimetry (TG), Contact Angle, Scanning Electron Microscopy (SEM), and water absorption experiments. The most significant balance of properties, within the analyzed content range (5, 7.5, and 10 wt.%), was obtained for the PA6/ABS-MA (10%) blend, indicating that even low concentrations of ABS-MA can improve the properties of PA6. Significant increases in impact strength and elongation at break have been achieved compared with PA6. The elastic modulus, tensile strength, HDT, and thermal stability properties of the PA6/ABS-MA blends remained at high levels, indicating that maleic anhydride interacted with amine end-groups of PA6. Torque rheometry, the Molau test, and SEM analysis suggested interactions in the PA6/ABS-MA system, confirming the high properties obtained. Additionally, there was a decrease in water absorption and the diffusion coefficient of the PA6/ABS-MA blends, corroborating the contact angle analysis.

## 1. Introduction

The term polymer blend describes the physical mixture of two or more polymers to develop new materials with distinct properties [1,2]. They represent a considerable share of polymer consumption worldwide and should increase even further over the next few years [3,4]. Developing polymer blends is one of the main strategies for producing new materials with different and improved properties from pure polymers. Additionally, blend preparation presents a lower cost when compared with the synthesis of new polymers [5]. Therefore, for the industrial sector, improving the mechanical properties of engineering polymers is more cost-effective through the preparation of polymer blends [6]. In general, polymer blend preparation has received considerable attention for toughening notch-sensitive thermoplastics, e.g., polyamide 6 (PA6) [7,8].

PA6 is a semicrystalline polymer with good chemical resistance in organic media, low melt viscosity, a high crystalline melting temperature, high tensile properties, and good impact strength when not notched, making it very attractive for engineering applications [9,10]. Common applications of PA6 are gears, seals, automotive parts, electrical components (circuit breakers, switches, and connectors), membranes, and biomaterials [11,12,13]. However, PA6 has some drawbacks, including high moisture absorption, dimensional instability, and fragile behavior when notched, creating restrictions on use in some applications [14,15,16]. Such deficiencies have been overcome by preparing PA6 mixtures with maleic anhydride (MA)-grafted polymers, such as ethylene-vinyl acetate (EVA-g-MA), styrene-butylene-ethylene-styrene (SEBS-MA), and ethylene-propylene-diene (EPDM-MA) [17,18,19]. These materials considerably increase PA6’s impact strength but compromise its elastic modulus, tensile strength, and heat deflection temperature (HDT) properties. Due to these limitations, the acrylonitrile-butadiene-styrene (ABS) terpolymer has emerged as a cost-effective alternative to improve PA6 properties [20,21].

ABS is considered an engineering thermoplastic that has a wide range of functional properties, such as high impact strength, good rigidity, chemical resistance, low density, dimensional stability, high surface brightness, thermomechanical stability, and ease of processing [22,23]. ABS incorporation can improve impact strength and reduce the water absorption level of PA6 and, therefore, PA6/ABS blends are of great technological interest [24]. However, this mixture is immiscible and incompatible, requiring the addition of a compatibilizing agent based on maleic anhydride (MA) to react with the amine end-groups of PA6 [25,26]. At the same time, there is a possibility of grafting maleic anhydride into the ABS chain, and, in this case, the reaction occurs directly between PA6 and ABS-g-MA [27,28].

Sun et al. [29] prepared ABS grafted with acrylic acid (AA), maleic anhydride (MA), and glycidyl methacrylate (GMA) for applications as impact modifiers of PA6. Subsequently, PA6/ABS blends functionalized with AA, MA, and GMA in a 70/30 wt.% proportion were extruded and injection-molded. The PA6/ABS blend (<100 J/m) showed a slight increase in impact strength compared to PA6. A considerable increase in impact strength (>800 J/m) was observed for the PA6/ABS-MA and PA6/ABS-GMA blends. The impact strength was even more pronounced with the ABS-MA addition. On the other hand, the PA6/ABS-AA blends showed an impact strength of about 220 J/m, indicating a low interaction of ABS-AA and PA6 compared to the ABS-MA and ABS-GMA modifiers. Transmission electron microscopy (TEM) analysis showed that the modified ABS is uniformly dispersed in the PA6 matrix, while the unmodified ABS tended to agglomerate.

Oliveira et al. [30] investigated the mechanical and thermomechanical properties of PA6/ABS blends (60/40 wt.%) compatibilized with styrene-maleic anhydride (SMA). The blend without the compatibilizer (PA6/ABS) showed an impact strength of about 36 J/m, lower than pure PA6 (40 J/m). In consequence, the PA6/ABS blend was considered brittle and incompatible. The simultaneous sequence of all PA6/ABS/SMA components (57.5/37.5/5 wt.%) showed an impact strength of 63.7 J/m (an increase of 59% compared to the PA6 matrix). This blend presented an elastic modulus of 2.3 GPa, which is a reduction of approximately 15% compared to PA6. At the same time, the HDT of PA6 (around 55 °C) increased by more than 20 °C with the addition of ABS, suggesting more structural stability.

Most of the works reported in the literature regarding PA6/ABS or PA/ABS-MA blends use ABS concentrations in the range of 30 to 50 wt.% [31,32,33,34]. However, there are practically no investigations concerning the processing of these blends using low ABS or ABS-MA concentrations, specifically 5 wt.%, 7.5 wt.%, and 10 wt.%. Hence, the development of PA6/ABS and PA6/ABS-MA blends with low ABS and ABS/MA concentrations constitutes a significant research gap, especially because the mechanical properties of PA6 can be tailored without severely compromising other properties.

Therefore, in this research, we studied the effect of low ABS and ABS-MA concentrations on the properties of PA6. The mechanical, thermomechanical, thermal, rheological, morphological, and water absorption kinetics properties were investigated and analyzed. Thus, with this detailed investigation, we expect to contribute to the database on polymer technology, which is paramount to both academic and industrial sectors.

## 2. Materials and Methods

### 2.1. Materials

Polyamide 6 (PA6) pellets supplied by Polyform (commercial code B300^®^, a density of 1.13 g/cm^3^, and a melt flow index of 2.9 g/10 min (235 °C/2.16 kg)) was used as the polymer matrix.

The acrylonitrile-butadiene-styrene (ABS) terpolymer (commercial code AE8000^®^, a density of 1.04 g/cm^3^, and a melt flow index of 5 g/10 min (220 °C/10 kg)) was used as an impact modifier and supplied as pellets by Innova. Table 1 presents the mechanical and thermomechanical properties of ABS.

Acrylonitrile-butadiene-styrene grafted with maleic anhydride (ABS-MA) (3.1% grafting degree) was used as an impact modifier. The chemical modification of ABS AE8000^®^ with maleic anhydride was carried out in our laboratory using an internal mixer. The detailed procedure for ABS grafting, the grafting degree, and the rheological, thermal, and spectroscopic properties can be found in the literature [35].

### 2.2. Methods

#### 2.2.1. Extrusion Processing

Before preparing the blends, polyamide 6 (PA6) was dried in a vacuum oven at 80 °C for 24 h. ABS and ABS-MA were dried in the vacuum oven at 60 °C for 24 h. The compositions (wt.%) of the PA6/ABS and PA6/ABS-MA blends are shown in Table 2.

**Figure 1:**
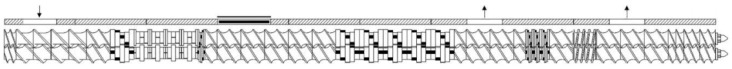
Extruder screw profile configured with distributive and dispersive elements [36].

The blends were dry mixed and subsequently processed in a Coperion Werner & Pfleiderer modular co-rotating twin-screw extruder, model ZSK (D = 18 mm and L/D = 40). The temperatures of all zones were kept at 230 °C, while the screw rotation speed was set at 200 rpm. A controlled feed rate of 2 kg/h was applied. The screw profile was configured with distributive and dispersive mixing elements, as shown in Figure 1. For comparative purposes, polyamide 6 (PA6) was processed and dried under the same conditions as the blends.

#### 2.2.2. Injection Molding

Before injection molding, the PA6 and the blends were dried in a vacuum oven for 24 h at 80 °C. Blends and PA6 were injection-molded in an Arburg Allrounder 207C Golden Edition injection machine to obtain the impact, tensile, and HDT specimens according to ASTM D256, ASTM D638, and ASTM D648 standards, respectively. The injection conditions of all specimens are presented in Table 3. After injection, specimens were stored and kept in a desiccator with silica for further characterizations.

### 2.3. Characterization of the Blends

Rheological curves were obtained in a Thermo Scientific Haake PolyLab QC mixer, with roller rotors, at 230 °C, and rotor speed of 60 rpm, under air atmosphere for 10 min.

The Molau test was performed by dissolving 0.5 g of pure PA6, pure ABS, ABS-MA, and the PA6/ABS and PA6/ABS-MA (90/10%) blends in 50 mL of formic acid (85%) under magnetic stirring for 1 h.

Fourier transform infrared spectroscopy (FTIR) was performed on a BRUKER Vertex 70 Spectrometer (attenuated total reflectance–ATR), in the range 4000 to 400 cm^−1^, with 32 scans and a resolution of 4 cm^−1^.

Scanning electron microscopy (SEM) analysis was carried out on the fracture surface of impact test specimens. A VEGAN 3 TESCAN scanning electron microscope, operating under vacuum with 30 kV voltage, was used. The fracture surfaces of the samples were subjected to gold sputtering before SEM analyses.

Izod impact strength tests were conducted on seven notched specimens (ASTM D256 standard) using a Ceast Resil 5.5 J device, operating with a 2.75 J hammer at room temperature (~23 °C).

Tensile tests were performed on injected specimens (ASTM D638 standard) using an EMIC DL 2000 universal testing machine with an elongation rate of 50 mm/min and a load cell of 20 kN at room temperature (~23 °C). The results were also analyzed for an average of seven specimens.

The heat deflection temperature (HDT) was obtained using Ceast HDT 6 VICAT equipment operating with a 1.82 MPa load and a heating rate of 120 °C/h (ASTM D648 standard). HDT was determined after the sample deflected 0.25 mm. The results presented are an average of three specimens.

Thermogravimetric (TG) analyses were conducted in a simultaneous TG/DSC TA Instruments SDT Q600 device, employing ~6 mg of each sample. The samples were heated from room temperature to 550 °C with a heating rate of 10 °C/min under a nitrogen atmosphere with a 100 mL/min flow rate.

Differential scanning calorimetry (DSC) scans were acquired using a TA Instruments DSC-Q20. The tests were performed under a nitrogen atmosphere (50 mL/min flow rate) using 6 mg samples. The samples were heated from 30 to 250 °C at a heating rate of 10 °C/min with an isotherm of 3 min.

Contact angle analysis was carried out through the sessile drop method using a portable contact angle Phoenix-i device, Surface Electro Optics–SEO model. A drop was deposited onto film samples using a micrometric doser. Images were captured and analyzed using the equipment’s software. The films were prepared using extruded beads in a hot press at 230 °C, applying 5 tons for 3 min. A square mold with a thickness of approximately 0.5 mm was used during compression molding.

The water absorption test was performed on injected specimens previously conditioned in a vacuum oven for 48 h at 80 °C. After 48 h in the vacuum oven, the samples were immediately weighed on a precision scale to obtain the mass before immersion (M_0_). Then, the specimens were completely submerged in water at room temperature (~23 °C) to measure the mass after immersion (M_t_). At predetermined intervals, the specimens were removed from the water, dried with a piece of cloth, weighed on a precision scale, and then placed back in the bath. The water absorption was calculated using Equation (1).

(1) $$\mathrm{Water}\;\mathrm{absorption}\;(\%)=\frac{M_{t}-{\;M}_{0}}{M_{0}}\;\times \;100\%$$

The diffusion coefficient (D) of PA6 and blends was determined using the methodology adopted by Vergnaud [37]. For short t times, D can be determined by Equation (2).

(2) $$\frac{M_{t}-M_{0}}{M_{\infty }}=\frac{2}{L}{\left(\frac{D}{\pi }\right)}^{1/2}t^{1/2}$$

The term M_t_ corresponds to the mass of water absorbed at time t, M_0_ is the mass of water absorbed at the initial instant, M_∞_ refers to the mass of water absorbed at equilibrium, and L corresponds to the thickness of the sample tested. A graph of (M_t_ − M_0_)/M_∞_ as a function of (t^1/2^)/L can be constructed for the various data obtained as a function of t. The diffusion coefficient is calculated from the angular coefficient (AC) and can be expressed by Equation (3):

(3) $$D=\frac{{\left(\mathrm{AC}\right)}^{2}\times \pi }{4}$$

## 3. Results and Discussion

### 3.1. Torque Rheometry

**Figure 2:**
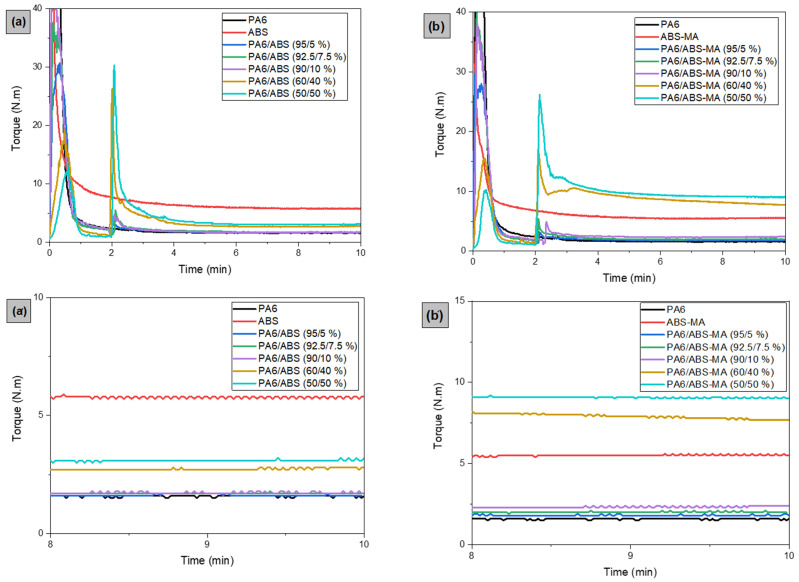
(**a**) Torque plots for PA6, ABS, and the PA6/ABS blends; (**b**) torque plots for PA6, ABS-MA, and the PA6/ABS-MA blends; (**a’**) an enlargement of (**a**); (**b’**) an enlargement of (**b**).

Toque rheometry was employed to evaluate the reactivity of the PA6/ABS and PA6/ABS-MA blends. Increments in torque values can be interpreted as interactions between chemical groups in the mixed polymers. Figure 2a,b presents the torque plots for PA6, ABS, and ABS-MA, as well as for the PA6/ABS and PA6/ABS-MA blends. After 2 min of processing, the torque of the pure polymers (PA6, ABS, and ABS-MA) remains constant, which indicates viscosity stability for the process conditions used. ABS showed the highest torque (5.8 N·m) at the end of processing, while PA6 and ABS-MA presented 1.6 N·m and 5.5 N·m, respectively.

ABS and ABS-MA were added to PA6 within 2 min after the test started, once the torque was stable. The PA6/ABS blends containing 5%, 7.5%, and 10% ABS presented torques of 1.6 N·m, 1.7 N·m, and 1.7 N·m, respectively. Compared to PA6, these samples did not increase or decrease the torque values (see Figure 2a), indicating no significant change in the viscosity profile of PA6 with low ABS concentration. We prepared PA6/ABS blends with 40% and 50% ABS to understand the influence of higher ABS contents in the PA6 matrix. The addition of 40% and 50% ABS increased the torque to 2.8 N·m and 3.2 N·m compared with PA6, respectively. Since torque is directly proportional to viscosity and the molar mass of the polymeric material, PA6/ABS (40% and 50%) blends have a higher viscosity than pure PA6. However, the torque values of PA6/ABS blends, regardless of the ABS content, were well below the toque results of pure ABS. This behavior indicates that there was no increase in the molar mass and chemical interaction between PA6 and ABS, which suggests an immiscible system [38]. The torque increase provided by additions of 40% and 50% ABS is exclusively due to the higher viscosity of pure ABS (additive effect).

Figure 2b shows a distinct behavior for PA6/ABS-MA blends containing 5%, 7.5%, and 10%. As the ABS-MA content is added and increased in PA6, torques of 1.8 N·m, 2 N·m, and 2.4 N·m are observed. The progressive torque increase suggests that the viscosity of the PA6/ABS-MA blends increased with ABS-MA content. This could indicate the formation of chemical bonds between polymers, forming a copolymer in situ [39]. Similar results were found by Choi et al. [40]. They evaluated the torque as a function of time for PA6/SMA (styrene-maleic anhydride) mixtures (7% MA) with different SMA contents (1.5 and 10 pcr). The authors observed the highest torque for the PA6/SMA (10 pcr) blend, indicating a reaction between maleic anhydride and the amine end-groups of PA6 to form an imide group.

**Figure 3:**
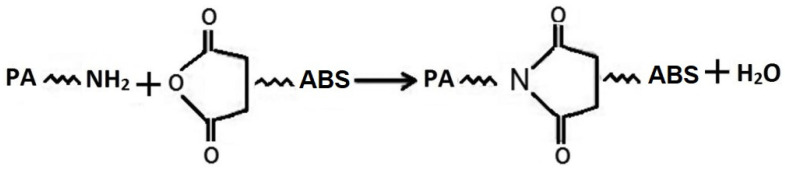
Reaction of maleic anhydride group of ABS-MA with PA6 amine group forming imide (adapted from [18]).

The addition and mixing of 40% ABS-MA with PA6 promoted a significant increase in torque to a value of 7.7 N·m. A more pronounced torque increment (approximately 9 N·m) occurred for the PA6/ABS-MA blend (50%). As a result, these blends showed increased viscosity, even surpassing the respective pure polymers (PA6 and ABS-MA). The torque of PA/ABS-MA (40 and 50%) blends supports the hypothesis of chemical reactions between the MA of ABS-MA and the amine end-groups in the PA6 chain, resulting in increased viscosity. Therefore, the torque increase for PA6/ABS-MA (5, 7.5, and 10%) blends is related to the chemical interaction between the functional groups, which contributed to the increased viscosity. As a result, the PA6/ABS-MA interface was strengthened, promoting compatibility (see mechanical properties and morphological results). Figure 3 illustrates a hypothetical formation scheme for PA6/ABS-MA blends. In general, blending occurs through a reaction between the amine end-groups of PA6 and the maleic anhydride group of ABS-MA.

### 3.2. Molau Test

**Figure 4:**
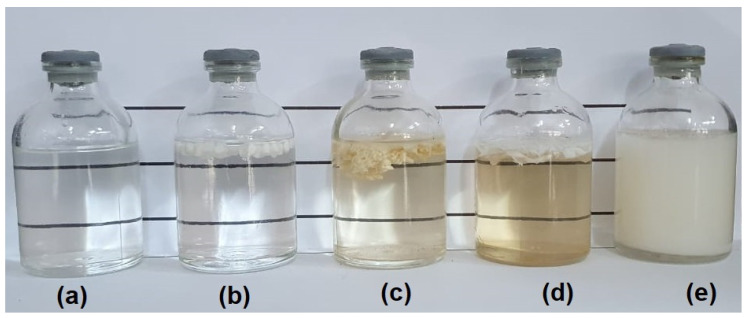
Molau test solutions in formic acid for (**a**) pure PA6; (**b**) ABS; (**c**) ABS-MA; (**d**) PA6/ABS (90/10%); (**e**) PA6/ABS-MA (90/10%).

The Molau test is the simplest method to characterize the compatibility of maleic anhydride-functionalized PA6 blends obtained by reactive extrusion [41]. The analysis is based on the difference in polymer solubility and allows the study of the formation of grafted copolymers in reactive mixtures. A polymer blend will form a milky solution if it reacts chemically in formic acid media, which depends on the emulsification of the reacted material [42]. Hence, the Molau test was applied to investigate the formation of a grafted copolymer between PA6 and ABS-MA. Figure 4 shows the Molau test for pure PA6, ABS, ABS-MA, and blends, respectively. The test was performed using PA6/ABS and PA6/ABS-MA blends with 90/10% proportions.

Pure PA6 was completely dissolved in formic acid, forming a clear solution, as shown in Figure 4a. Lin et al. [43] observed similar behavior for polyamide 6. On the other hand, ABS and ABS-MA proved to be insoluble since they precipitated on the surface. ABS and ABS-MA exhibited a low dissolution level, with only superficial softening and pellet agglomeration. This happened because the solution is acidic. The PA6/ABS blend presented a cloudy solution and a high level of ABS precipitation as agglomerated pellets. Such behavior indicates the absence of a reaction in the PA6/ABS system. It was observed that the PA6/ABS-MA blends formed a milky suspension when dissolved in formic acid, suggesting that there were chemical reactions between PA6 and ABS-MA, confirming the torque rheometry results. The white solution is formed due to polyamide 6 dissolution in formic acid, dragging the ABS-MA that reacted during processing. Wang et al. [44] observed similar behavior for PA6/poly (ethylene-1-octene) (POE) blends functionalized with maleic anhydride.

**Figure 5:**
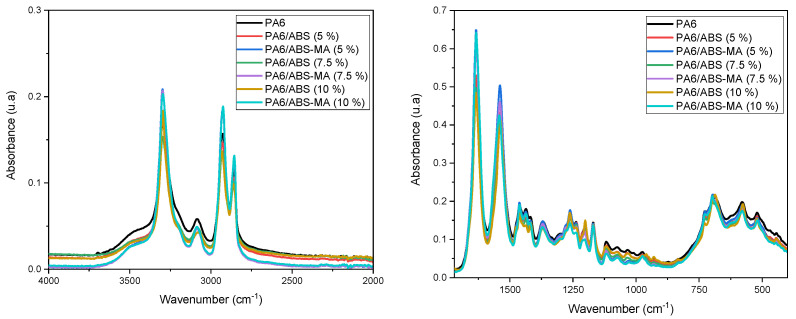
FTIR spectra of pure PA6 and the blends, respectively.

Fourier transform infrared spectroscopy (FTIR) analysis was performed to verify the interaction between PA6/ABS and PA6/ABS-MA, as shown in Figure 5.

Pure PA6 and the blends, regardless of ABS and ABS-MA content, showed an intense band at 3295 cm^−1^ due to the stretching vibration of N-H groups. In addition, the bands at 2930 cm^−1^ (asymmetric) and 2852 cm^−1^ (symmetric) appeared to correspond to C-H bonds. The absorption band at 1633 cm^−1^ corresponds to the stretching vibration of the C=O group (Amide I). The band at 1539 cm^−1^ refers to the bending vibration of the N-H group and the stretching vibration of C-N groups (Amide II) [12,45,46]. In general, the PA/ABS and PA6/ABS-MA blends showed spectra similar to PA6. However, the characteristic N-H and C=O bands were more intense for PA6/ABS-MA blends. Such behavior suggests a reaction between PA6 and ABS-MA, as indicated by torque rheometry and the Molau test. When mixing ABS-MA and PA6, the amine end groups possibly reacted with the maleic anhydride groups of ABS-MA. As a result, N-H and C=O functional groups formed and their bands overlapped with the characteristic absorption bands of PA6, leading to the observed increased intensity.

### 3.3. Scanning Electron Microscopy (SEM)

**Figure 6:**
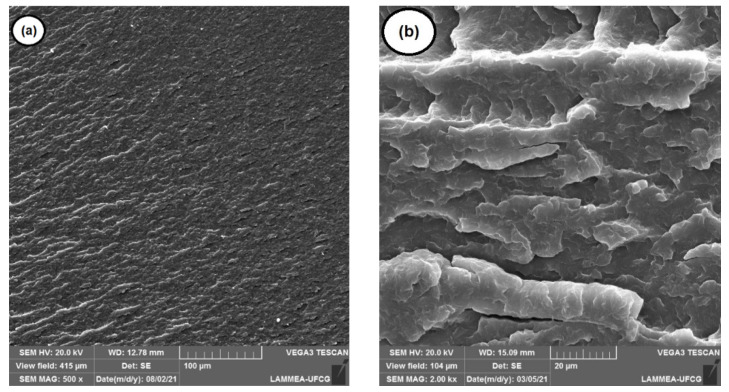
SEM micrographs showing the morphology on the fracture surface of pure PA6: (**a**) 500× magnification; (**b**) 2000× magnification.

Figure 6a,b shows the SEM micrographs of pure PA6, with a magnification of 500× and 2000×, respectively. As noted, the fracture surface of the pure PA6 showed a regular ductile fracture appearance, a behavior similar to that found in the literature [47].

**Figure 7:**
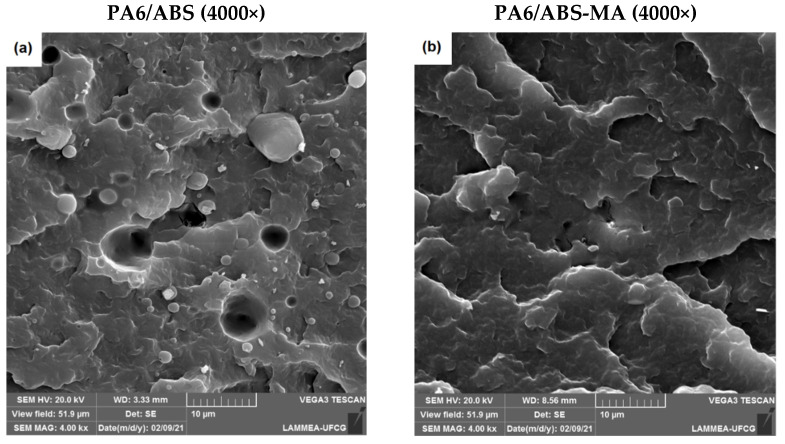

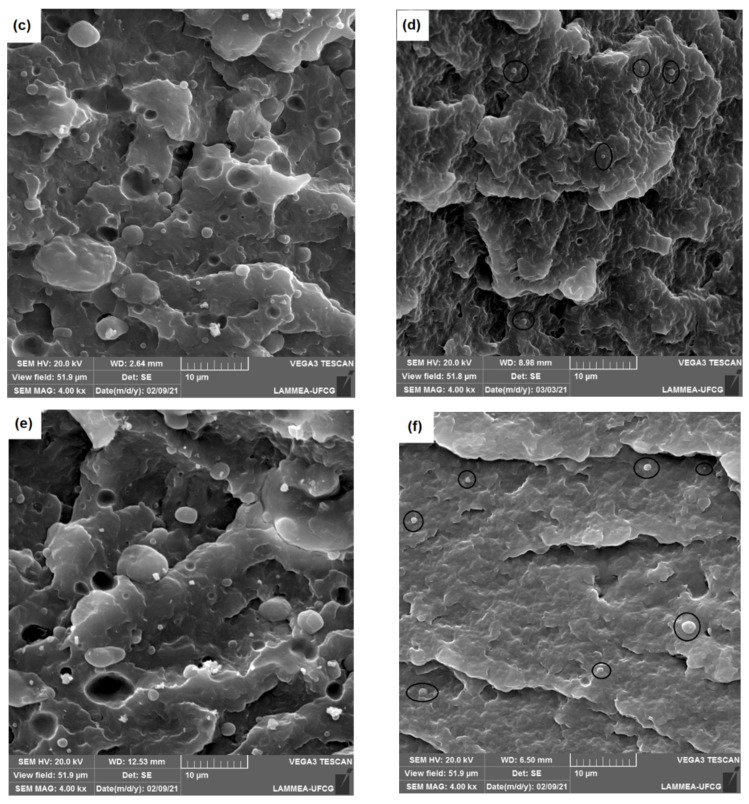
SEM micrographs of the fractured surfaces of (**a**) PA6/ABS (5%); (**b**) PA6/ABS-MA (5%); (**c**) PA6/ABS (7.5%); (**d**) PA6/ABS-MA (7.5%); (**e**) PA6/ABS (10%); (**f**) PA6/ABS-MA (10%), respectively.

The mechanical properties are strongly influenced by morphology, and, therefore, scanning electron microscopy (SEM) was used to analyze the morphology of the blends. Figure 7 shows SEM micrographs of PA6/ABS and PA6/ABS-MA blends with various ABS and ABS-MA contents.

The PA6/ABS blends presented a biphasic structure with spherical ABS particles of varying sizes, close to each other, dispersed in the PA6 matrix. The morphology of PA6/ABS blends has many empty spaces, indicating that ABS particles have been pulled out of the PA6 matrix during the impact test. Besides, the presence of PA6/ABS interfaces without any adhesion suggests low interfacial resistance. The fracture aspect of PA6/ABS blends clearly shows a poor adhesion between the dispersed phase (ABS) and the continuous phase (PA6), characterizing an incompatible system. As ABS content increased in the PA6/ABS blends, the particles tended to coalesce. Majumdar et al. [48] prepared PA6/ABS blends using PA6 with four different molar masses. The authors observed that the PA6 blends with the lowest viscosity had large ABS domains, attributed to the different viscosities of PA6 and ABS. Therefore, ABS domains can coalesce at high temperatures when PA6 has very low viscosity, resulting in the loss of mechanical properties.

The ABS functionalization process by maleic anhydride produced a stabilizing effect on the morphology of PA6/ABS-MA blends, as seen in Figure 7b,d,f. It was observed that practically no ABS-MA domains were pulled out from the PA6 matrix, evidencing an interfacial strengthening, as discussed in torque rheometry. Additionally, there was a significant reduction in the average particle size of ABS-MA, as well as a substantial improvement in adhesion between PA6 and ABS-MA phases (black circle). The morphology of PA6/ABS-MA blends indicates a synergistic interaction between the maleic anhydride group of ABS-MA and the PA6 amine group, contributing to reducing interfacial tension and avoiding particle coalescence [49]. Consequently, there was a significant reduction in the dispersed phase (ABS-MA) size, which is important for the mechanical properties [50], especially the impact strength. This is because smaller dispersed-phase particles increase the energy dissipation in PA6/ABS-MA blends.

The micrographs of PA6/ABS blends highlight the incompatibility of this system. There is weak interfacial adhesion and lack of wetting between PA6 and ABS phases, corroborating the low values obtained in the mechanical properties. However, the ABS grafting procedure with maleic anhydride resulted in a higher level of interaction with PA6, as shown in the morphology. The improvement in interfacial adhesion and the dispersed phase (ABS-MA) refinement contributed to an increase in the mechanical performance of PA6/ABS-MA blends.

### 3.4. Izod Impact Strength

**Figure 8:**
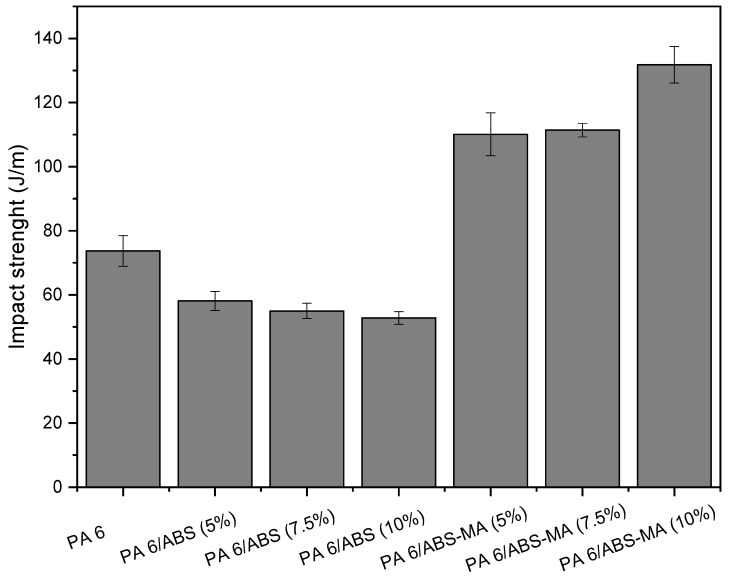
Impact strength of PA6 and binary blends.

Figure 8 shows the impact strength results of PA6 and the binary blends as a function of ABS and ABS-MA content, respectively.

Despite being an engineering polymer, PA6 does not have high impact strength (73.7 J/m) when notched. The PA6/ABS blends showed impact strength values lower than PA6. Although ABS has a high impact strength (Table 1), it did not act as an impact modifier for PA6. There is a tendency towards progressive impact strength reduction with an ABS increase, suggesting an intensification of a fragile behavior. These results demonstrate the incompatibility of the PA6/ABS system, corroborating with the SEM morphology obtained.

PA6/ABS-MA showed a considerable increase in impact strength compared with pure PA6 and PA6/ABS blends. The increase in impact strength reveals that ABS-MA addition promoted interactions between maleic anhydride groups and amine end-groups of PA6, as suggested by torque rheometry and Molau test results. In this case, the interface is strengthened (as observed in the SEM analysis), which is essential for energy transfer and dissipation between phases [51].

PA6/ABS-MA blends containing 5% and 7.5% ABS-MA increased the impact strength by approximately 50% compared with PA6. However, the impact strength of these two compositions is similar, considering experimental error margins. A very significant increase of 79% in impact strength (compared with pure PA6) is presented by the PA6/ABS-MA (10%) blend. These results indicate that ABS-MA acts as a toughening agent for PA6, and that reactive compatibility has occurred [52]. Microvoids would develop at the interface if there were no interactions within the PA6/ABS-MA system. This would lead to crack formation and easier crack propagation, resulting in low impact strength, as seen for PA6/ABS blends. In general, low concentrations of ABS-MA can improve the PA6 impact strength. Thus, it is possible to expand the application range for products that require impact strength greater than 120 J/m.

### 3.5. Tensile Properties

**Figure 9:**
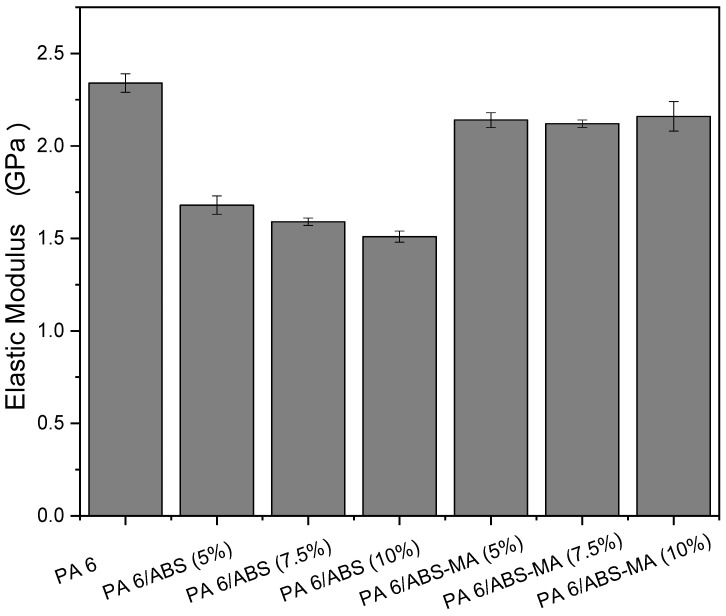
Tensile modulus of elasticity of PA6 and its binary blends.

Figure 9 shows the elastic modulus of PA6 and its binary blends as a function of the ABS-MA and ABS content. Polyamide 6 showed the highest elastic modulus (~2.4 GPa) and, thus, higher stiffness among all samples. The PA6/ABS-MA and PA6/ABS blends presented reduced elastic modulus compared with pure PA6. Such behavior is due to the flexibility given by polybutadiene in the ABS structure. However, compared with PA6, the PA6/ABS-MA blends did not suffer a drastic reduction in elastic modulus. It was found that a higher ABS-MA content did not seem to influence the elastic modulus of PA6/ABS-MA blends since these values are close when considering the experimental error margin. Contrastingly, a marked elastic modulus reduction occurred when the non-functionalized ABS was added, due to the low interfacial adhesion, as verified in SEM analysis. The PA6/ABS blends showed a decreasing tendency in elastic modulus with the ABS increase. By comparison, the PA6/ABS-MA blends showed higher elastic modulus performance than PA6/ABS blends. Such a behavior can be attributed to the increased level of interaction within PA6/ABS-MA blends, with reactions between maleic anhydride and amine groups of PA6 (see torque rheometry and Molau test results), contributing to an improved synergistic effect in this property [53].

**Figure 10:**
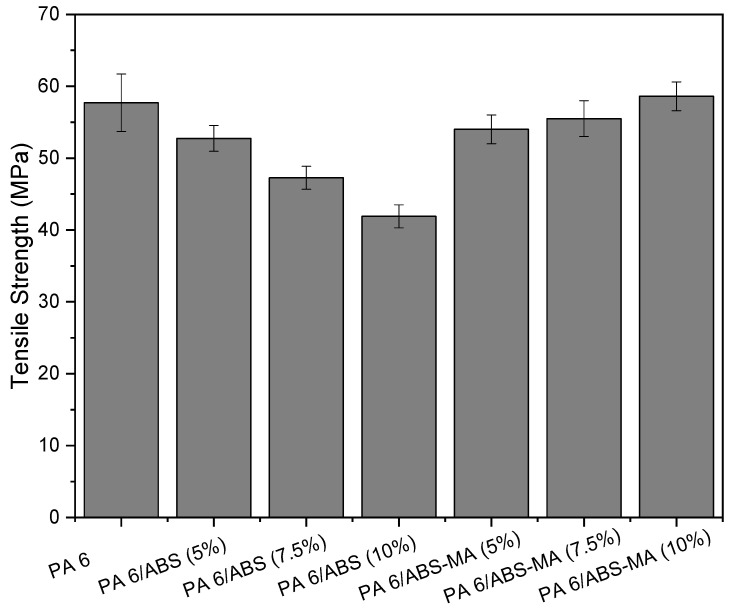
Tensile strength of PA6 and its binary blends.

Figure 10 shows the tensile strength results of PA6 and the PA6/ABS-MA and PA6/ABS blends, respectively. The tensile strength of PA6/ABS blends decreased (compared to pure PA6) with increasing ABS content. In this case, the PA6/ABS blends deform at lower stresses. On the contrary, there was a continuous increase in the tensile strength of PA6/ABS-MA blends, especially for 10% ABS-MA, which reached strengths comparable with PA6. The higher ABS-MA content seemed to have a positive influence on this property. The results show that ABS-MA interacts with the PA6 matrix, improving the PA6 deformation mechanism even at low concentrations. Tensile strength is a property measured in the plastic regime, and it is, therefore, affected by the degree of interfacial adhesion. If there were no interaction, the dispersed phase would act as a stress concentration point, reducing resistance, as verified for PA6/ABS blends.

**Figure 11:**
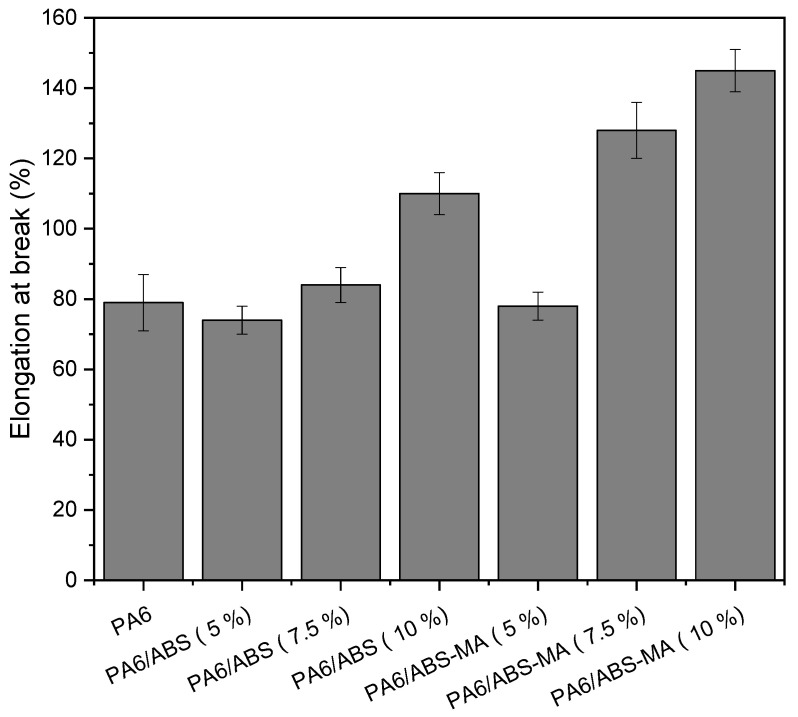
Elongation at break of PA6 and blends as a function of ABS and ABS-MA concentrations.

Figure 11 shows the elongation at break of pure PA6 and the blends with different ABS and ABS-MA contents.

The addition of 5% and 7.5% ABS did not significantly improve the elongation at break of PA6. The same pattern was observed for the PA6/ABS-MA blend with 5% ABS-MA. For 10% ABS content, the elongation at break tended to increase, suggesting that a low concentration does not cause ductility to deteriorate under tensile stress. However, the PA6/ABS-MA blends with 7.5% and 10% ABS-MA showed improved deformation mechanism and high ductility. The PA6/ABS-MA blends (10%) achieved the highest deformation (~142%), suggesting the greatest synergistic effect for this property. Such behavior indicates that ABS-MA promoted interactions with PA6, supporting the torque rheometry, Molau, and FTIR tests.

**Figure 12:**
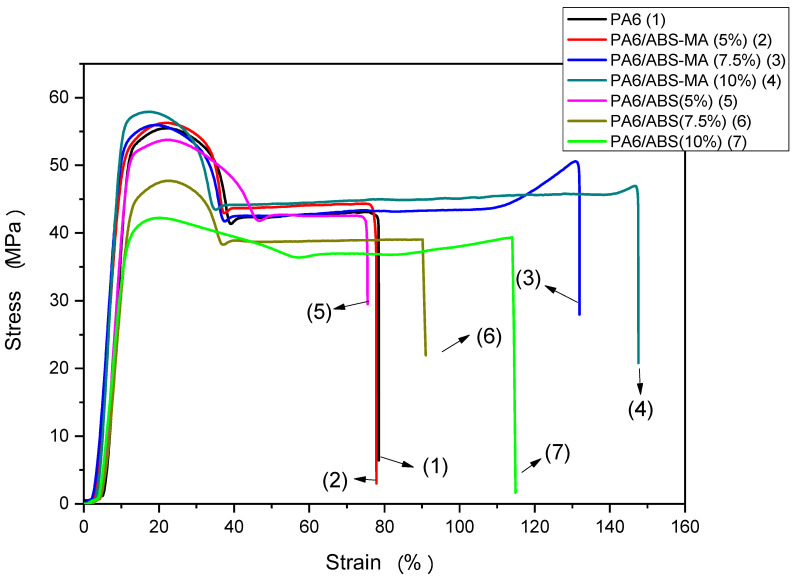
Stress–strain plots of PA6 and the PA6/ABS-MA and PA6/ABS blends, respectively.

Figure 12 shows the stress–strain curves of PA6 and PA6/ABS-MA and PA6/ABS blends, respectively. There was a continuous reduction in the yield strength of PA6/ABS blends with ABS content and in comparison with the PA6 matrix and the PA6/ABS-MA blends. The yield strength of the PA6/ABS-MA blends did not decrease or increase when compared with PA6. This behavior is related to the ABS-MA functionality that creates better interfacial adhesion (see SEM results). Consequently, the dispersed ABS-MA deformed with the matrix and promoted a synergistic effect favoring the deformation mechanism.

PA6/ABS blends with 5% and 7.5% ABS did not significantly improve the elongation at break compared with PA6. However, the value found for the PA6/ABS (10%) blend was higher than that of the matrix. The PA6/ABS (10%) blend showed ductile behavior under tensile stress, which did not occur for the impact strength test. This is probably due to the different load speeds of the tests. For high load speeds, such as in the impact test, ABS particles non-functionalized with maleic anhydride act as defects since they displayed low adhesion in the PA6/ABS blends (see SEM results). However, tensile tests have low load speeds, and so cracks propagate more slowly [54]. The PA6/ABS-MA blends showed a tendency to improve the elongation at break property with the addition and increments of ABS-MA. The PA6/ABS-MA blends with 7.5% and 10% showed a considerable elongation increment, while the composition with 5% ABS-MA showed similar elongation to PA6. The PA6/ABS-MA (10%) blend reached a deformation of 141%, indicating the highest synergistic effect and reinforcing the hypothesis that ABS-MA suffered deformation with the PA6 matrix.

### 3.6. Heat Deflection Temperature (HDT)

**Figure 13:**
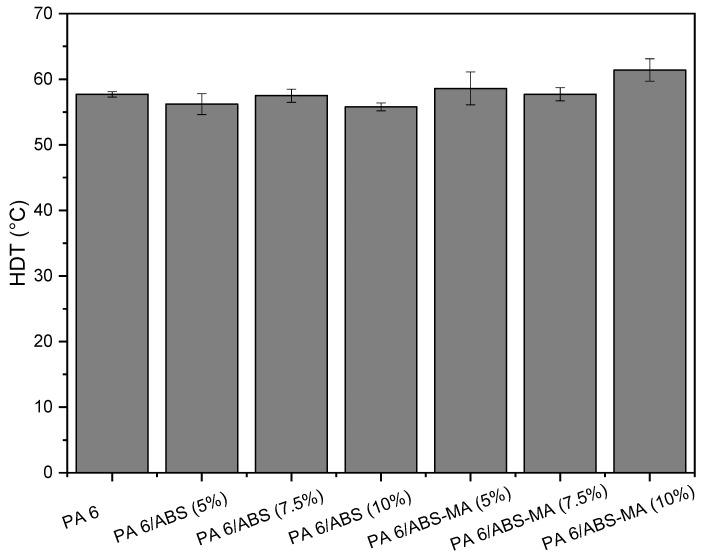
Heat deflection temperature of PA6 and its binary blends as a function of ABS-MA and ABS content, respectively.

The heat deflection temperature (HDT) test is extremely important in the polymer industry as it simulates the polymer’s behavior at temperatures above ambient [55,56]. Figure 13 shows the heat deflection temperature results for PA6 and its binary blends as a function of ABS-MA and ABS content, respectively.

The HDT value for pure PA6 was around 57.7 °C. The PA6/ABS blends showed HDT values in the range of 56 to 57.5 °C, while for PA6/ABS-MA systems, the results were between 57.8 and 61.4 °C. There were no statistically significant differences in HDT between pure PA6 and blends. Therefore, the thermomechanical resistance was similar. The HDT values of the blends, regardless of using ABS or ABS-MA, are very close to that obtained for PA6. The addition of ABS as a PA6 impact modifier, compared with other elastomeric impact modifiers (SEBS-MA and EPDM-MA), has the advantage of not lowering the heat deflection temperature (HDT). The increase or preservation of thermomechanical resistance is attributed to the presence of the SAN phase, as reported by Oliveira et al. [26].

From a technological perspective, the HDT result observed for the PA6/ABS-MA (10%) blend is important, as it slightly increased compared to the matrix. Furthermore, contrary to PA6, the tensile properties were similar, with similar stiffness and high impact strength of 131.9 J/m. Based on these results, mixing PA6 with ABS-MA (10%) promoted the toughening of PA6 at room temperature (~23 °C).

### 3.7. Differential Scanning Calorimetry (DSC)

**Figure 14:**
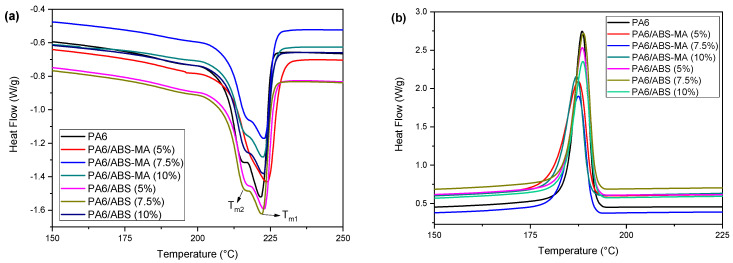
DSC curves of PA6 and its blends: (**a**) Crystalline melting temperature and (**b**) crystallization temperature.

Table 4 provides the melting and crystallization parameters, as well as the degree of crystallinity of PA6 and its binary blends. Figure 14 shows the DSC curves obtained during the second heating cycle. According to Table 4, PA6 has two melting temperature peaks (T_m1_ and T_m2_), attributed to the crystalline phases α and γ [57], which have melting temperatures of 222.1 °C and 212.8 °C, respectively. The melting temperatures of the blends, T_m1_ and T_m2_, did not show significant differences compared with pure PA6. However, it is possible to observe the disappearance of the Tm_2_ melting peak for the PA6/ABS-MA (5%) blend in Figure 14a. Therefore, adding ABS-MA (5%) probably affected the crystallinity of PA6 and contributed to the disappearance of the less thermally stable γ phase.

The PA6/ABS and PA6/ABS-MA blends showed a reduction in melting enthalpy (∆H_f_) compared with PA6, requiring less energy to melt the crystals. This behavior is due to the addition of amorphous components, ABS and ABS-MA, which contribute to reducing the energy required for crystal melting. The crystallization temperatures of the blends essentially did not change compared with the characteristic peak of PA6. However, the crystallization peaks of PA6/ABS-MA blends decreased in intensity compared with pure PA6 and PA6/ABS blends, as shown in Figure 14b. In this case, PA6/ABS-MA blends require less energy to promote crystallization.

The degree of crystallinity (X_c_) of the PA6/ABS and PA6/ABS-MA blends is lower than that of the PA6 matrix. This indicates that the presence of ABS and ABS-MA terpolymers might have inhibited the crystallization of PA6. Amorphous materials in the matrix hinder crystal formation and packaging, reducing crystallinity. The addition of ABS-MA in PA6 reduced the degree of crystallinity more severely, mainly for compositions containing 7.5% and 10%, compared with PA6/ABS blends. This finding reinforces the hypothesis that chemical interactions occurred between maleic anhydride and the amine end-groups of PA6, increasing viscosity in the system, as seen in torque rheometry. In general, a viscosity increase reduces molecular mobility and the probability of forming stable nuclei that grow into crystals [59].

**Figure 15:**
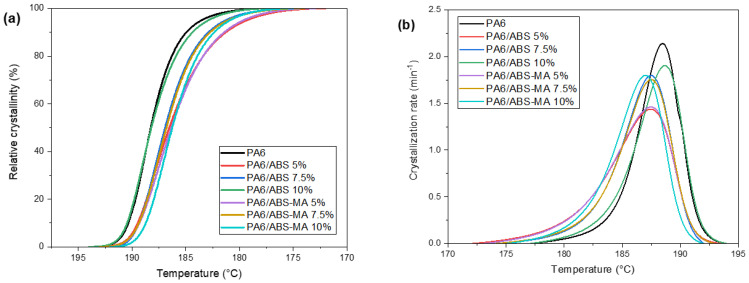
(**a**) Crystallization rate; (**b**) crystalline fraction.

Figure 15a,b shows the relative crystallinity and crystallization rate of pure PA6 and blends, respectively. The relative crystallinity curves of pure PA6 and its blends had a sigmoidal shape, suggesting phase transformation [60,61]. Compared with other formulations, the crystallization process started at higher temperatures for pure PA6 and PA6/ABS (10%) blend. Such behavior implies early crystallization during cooling, accelerating the injection-molding cycle. Moreover, PA6 and the PA6/ABS (10%) blend showed faster crystallization, as evidenced by the crystallinity rate (Figure 15b).

**Figure 16:**
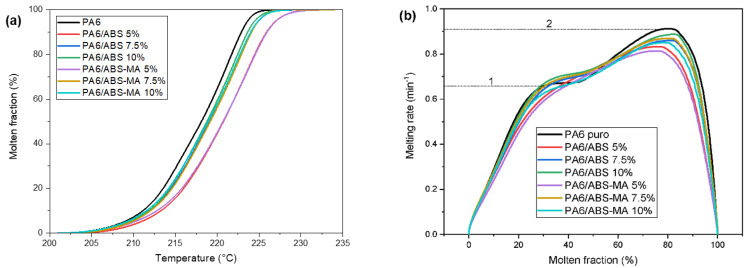
(**a**) Molten fraction of crystals; (**b**) melting rate as a function of the molten fraction of crystals.

Figure 16a,b shows the behavior of the molten fraction and the melting rate as a function of the molten fraction of the crystals, respectively. Regarding the molten fraction, PA6 started to melt at a lower temperature, suggesting the existence of larger amounts of smaller or less-perfect crystals [62,63]. The PA6/ABS (5%) and PA6/ABS-MA (5%) blends behaved similarly, with a curve displacement to higher temperatures. The increase in ABS and ABS-MA content to 7.5% and 10% essentially did not affect the crystalline molten fraction behavior as seen by the superimposed curves. Figure 16b indicates the formation of heterogeneous crystals with different crystalline melting rates (1 and 2). The small shoulder between 30 and 40 °C can be attributed to the existence of smaller crystals. Between 80 and 100 °C, a more intense peak appeared for PA6 and blends, suggesting the presence of more thermally stable crystals. The PA6/ABS (5%) and PA6/ABS-MA (5%) blends did not present such shoulders between 20 and 40 °C, indicating that, for these compositions, the less-perfect crystals are in smaller quantities. Consequently, the molten fraction shifted to higher temperatures, as seen in Figure 16a.

### 3.8. Thermogravimetry (TG)

**Figure 17:**
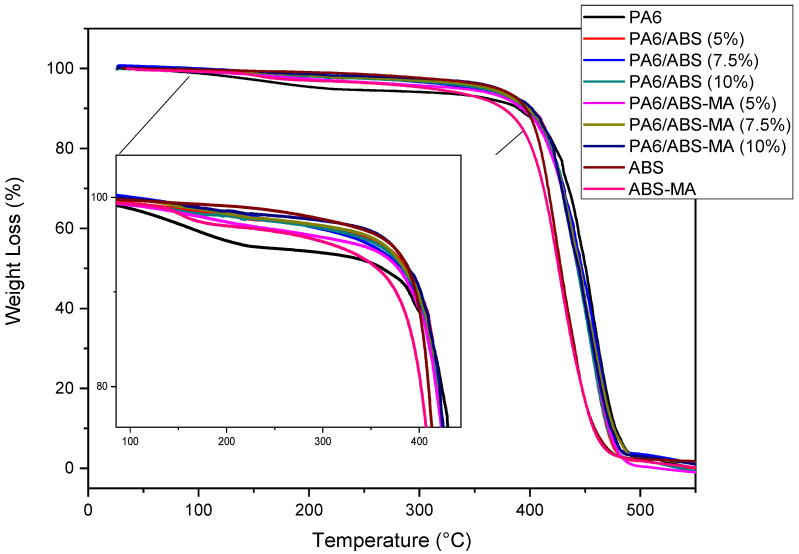
TG curves for pure PA6 and its binary blends.

Figure 17 shows the thermal stability behavior of PA6, PA6/ABS-MA, and PA6/ABS blends. Pure PA6 shows a slope in the TG curve between 60 and 200 °C, possibly due to humidity [64]. In this temperature range, the PA6/ABS-MA and PA6/ABS blends showed higher thermal stability than PA6. The addition of ABS-MA and ABS promoted the formation of a barrier effect that reduced moisture absorption, positively influencing thermal stability. This finding corroborates the higher contact angles for PA6/ABS-MA and PA6/ABS blends presented later.

Pure PA6 and PA6/ABS-MA and PA6/ABS blends showed only one decomposition stage (Figure 17), with essentially 100% of the entire mass consumed. The decomposition is associated with polymeric chains, which undergo bond-splitting processes. From 420 °C, the PA6 curve shifts to higher temperatures, indicating superior thermal stability compared with pure ABS and ABS-MA. The PA6/ABS-MA and PA6/ABS blends exhibit thermal decomposition behavior intermediate between those of the pure components. However, regardless of the composition, the thermal stability of the blends hardly changed compared with the PA6 matrix.

The PA6/ABS blends should have thermal stability higher than PA6/ABS-MA blends since non-functionalized ABS is more thermally stable. However, such behavior did not occur, which is probably due to the weak interactions between ABS and PA6 in the mixture, as seen in the mechanical results. On the other hand, the PA6/ABS-MA blends show evidence of a synergistic interaction between the components, with a stabilizing effect. Again, there is evidence of reactivity between PA6 and ABS-MA, increasing the interfacial interactions, stabilizing the morphology (see SEM results), and promoting higher compatibility.

### 3.9. Contact Angle

**Figure 18:**
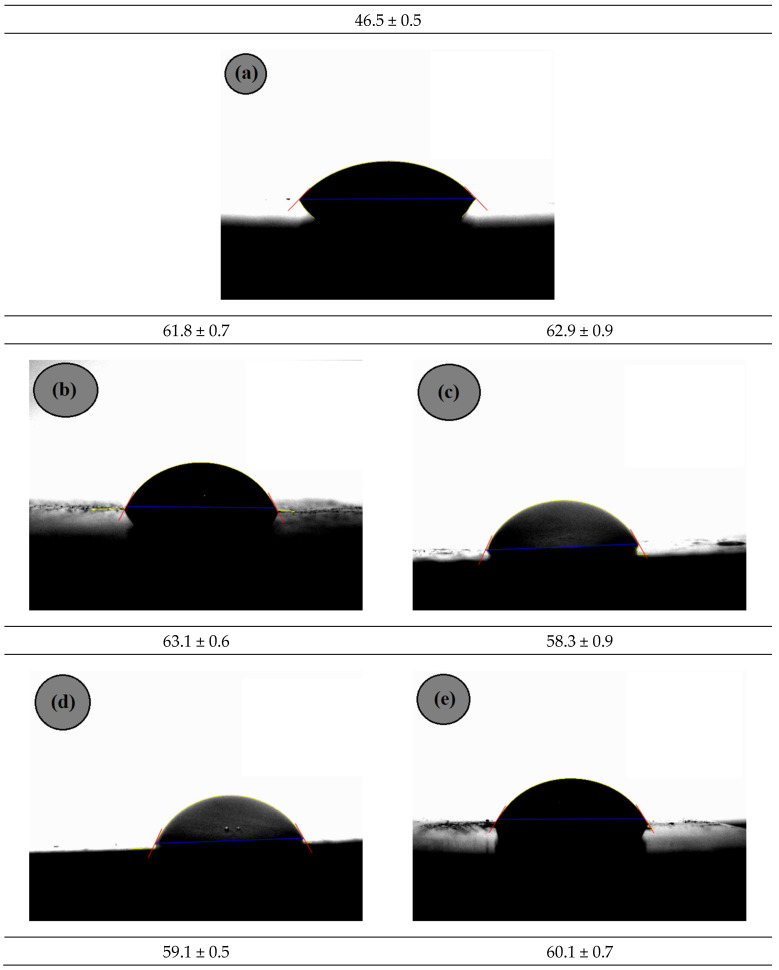

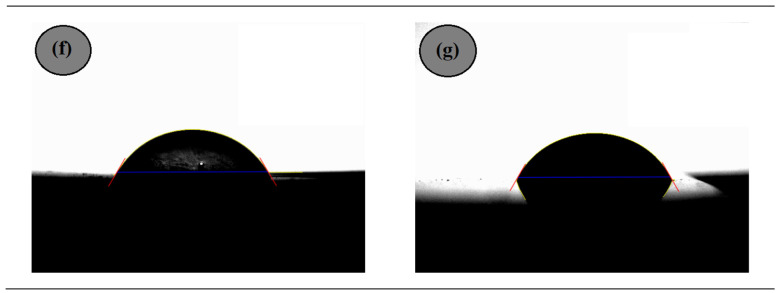
Mean contact angle at 200 s for (**a**) PA6; (**b**) PA6/ABS-MA (5%); (**c**) PA6/ABS-MA (7.5%); (**d**) PA6/ABS-MA (10%); (**e**) PA6/ABS (5%); (**f**) PA6/ABS (7.5%); (**g**) PA6/ABS (10%).

Figure 18 shows the results of the contact angle analysis for PA6 and its ABS and ABS-MA blends.

PA6 had the smallest contact angle (46.5°), suggesting greater water affinity, i.e., higher hydrophilic character. This is due to the molecular structure of PA6, which presents hydrogen bonds between carbonyls and the hydrogen of the amide group, making it hygroscopic. The PA6/ABS and PA6/ABS-MA blends, regardless of the dispersed phase content, had a higher contact angle than pure PA6, i.e., the addition of ABS and ABS-MA reduced the hydrophilic character of PA6. These materials have butadiene and styrene as non-polar parts of their structures, which reduces the interaction with water. Therefore, this justifies the increased contact angle of the blends. The contact angle results are important since one of the limitations of PA6 is its water affinity. In this case, even low concentrations of ABS-MA and ABS produced a decrease in the hydrophilic character of PA6.

PA6/ABS-MA blends generally have contact angles higher than PA6/ABS blends. This is because maleic anhydride reacts with the amine groups of PA6, contributing to better-dispersing ABS-MA, as seen in the SEM images. On the other hand, PA6/ABS blends presented a phenomenon of coalescence of ABS dispersed particles, decreasing the protection against the effect of the liquid and, consequently, providing a lower contact angle for PA6/ABS blends.

### 3.10. Water Absorption Kinetics

**Figure 19:**
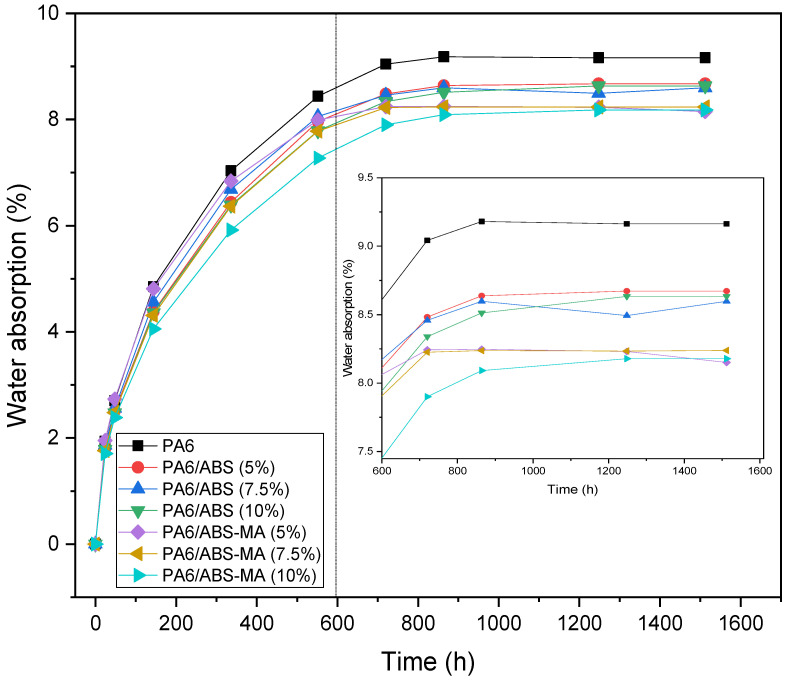
Water absorption on PA6 and its ABS and ABS-MA blends.

Figure 19 shows the water absorption behavior of pure PA6 and its ABS and ABS-MA blends. PA6 and its blends showed rapid water absorption kinetics at the beginning of the experiment. The high water absorption is attributed to the hydrophilic nature of the amide groups, which favors hydrogen bonds with water [65,66]. After 600 h, the curve undergoes a smooth change in slope, reaching a linear and stable level. This represents the level of humidity saturation. The PA6/ABS and PA6/ABS-MA blends presented lower water absorption compared with pure PA6, therefore indicating their hydrophobic behavior and confirming the contact angle results. Maleic anhydride-functionalized ABS has a higher tendency to reduce the water absorption of PA6 due to the higher interaction and distribution of the dispersed phase. As a result, there was a higher barrier effect against water diffusion in the PA6 matrix.

**Figure 20:**
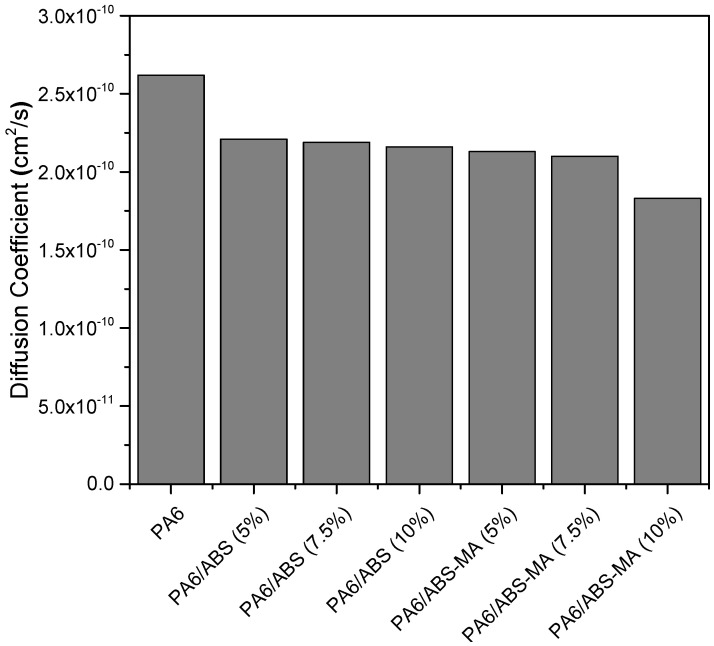
Diffusion coefficients of PA6 and its blends.

Figure 20 presents the diffusion coefficient results of PA6 and its ABS and ABS-MA blends.

The diffusion coefficient (D) corresponds to the ability of water to diffuse through the material. Pure PA6 showed the highest diffusion coefficient, i.e., the water mobility in its structure was the highest, which is ascribed to the higher hydrophilic characteristic observed in the contact angle result. In comparison with PA6, the addition of ABS and ABS-MA reduced the diffusion coefficient of all blends. This indicates a reduction in the water migration rate through the samples, which is more evident for the PA6/ABS-MA blends. Therefore, even for low ABS and ABS-MA concentrations, there was a barrier effect tendency, hampering water mobility.

## 4. Conclusions

An investigation on the tailoring of PA6 properties was conducted using low concentrations of ABS and ABS-MA. The development of PA6/ABS-MA blends has proven to be effective, as it showed considerably higher impact strength and elongation at break than pure PA6 and PA6/ABS blends. The elastic modulus and tensile strength properties of PA6/ABS-MA blends are comparable with pure PA6. This indicates that the addition of ABS-MA in small amounts toughens PA6 without compromising its stiffness. The torque rheometry and the Molau test of PA6/ABS-MA blends suggested interactions between PA6 and ABS-MA, justifying the good mechanical properties. The PA6/ABS and PA6/ABS-MA heat deflection temperatures (HDT) and thermal stability were practically unchanged compared with PA6. The crystalline melting temperature and crystallization parameters of the blends did not change compared with PA6. According to contact angle and water absorption analyses, PA6/ABS and PA6/ABS-MA blends presented a reduced water interaction. This investigation is important, given that one of the highest drawbacks of PA6 is its water affinity. Even at low ABS-MA concentrations (5, 7.5, and 10%), there was higher resistance to moisture absorption. Scanning electron microscopy (SEM) indicated that the PA6/ABS-MA blends have a refined and stable morphology, producing materials with good mechanical, thermal, and thermomechanical properties. The optimal properties were achieved with the PA6/ABS-MA (10%) blend, which indicates that even low-concentration ABS-MA can improve and tailor PA6 properties. The developed blends show potential for application in gears, dies, tubes, billets, plates, and rollers.

## Figures and Tables

**Table 1:** Data provided by the manufacturer of ABS AE8000^®^.

Properties	Standard	Results
Izod impact strength	ASTM D-256	392 J/m
Heat deflection temperature	ASTM D-648	86 °C
Flexural strength	ASTM D-790	69 MPa
Flexural modulus of elasticity	ASTM D-790	2260 MPa
Tensile strength	ASTM D-638	43 MPa

**Table 2:** Binary blends compositions.

Nomenclature	PA6 (wt.%)	ABS (wt.%)	ABS-MA (wt.%)
PA6	100	-	-
PA6/ABS	95	5	-
PA6/ABS	92.5	7.5	-
PA6/ABS	90	10	-
PA6/ABS-MA	95	-	5
PA6/ABS-MA	92.5	-	7.5
PA6/ABS-MA	90	-	10

**Table 3:** Injection-molding conditions.

Parameters	Conditions
Injection pressure (bar)	1200
Temperature profile (°C)	230, 240, 240, 240, 245
Mold temperature (°C)	50
Cooling time in mold (s)	25
Holding pressure (bar)	700

**Table 4:** Melting and crystallization parameters of PA6 and its blends: PA6/ABS and PA6/ABS-MA.

Samples	T_m_ (°C)		ΔH_f_ (J/g)	T_c_ (°C)	X_c_ (%)
	T_m1_	T_m2_			
PA6	222.1	212.8	56.4	188.5	30.0
PA6/ABS (5%)	223.4	213.1	50.1	188.5	28.1
PA6/ABS (7.5%)	222.8	212.9	51.4	188.8	29.5
PA6/ABS (10%)	222.7	212.5	45.5	188.7	26.7
PA6/ABS-MA (5%)	224.1	-	49.4	187.4	27.7
PA6/ABS-MA (7.5%)	223.2	214.9	42.7	187.5	24.6
PA6/ABS-MA (10%)	223.3	214.5	41.8	187.2	24.7

T_m_ = Melting peak temperature; ∆H_f_ = Melting enthalpy; X_c_ = Degree of crystallinity, calculated by X_c_ = ($\frac{{\Delta H}_{f}}{W_{\mathrm{PA}6}\times {\;\Delta H}_{f\;100\%}}$) × 100%, where ∆H_f 100%_ = Melting enthalpy of polyamide 6 with 100% crystallinity, 190.8 J/g [58].

## Data Availability

Not applicable.
